# The Influence of Obesity on Cognitive Functioning Among Healthcare Professionals: A Comprehensive Analysis

**DOI:** 10.7759/cureus.42926

**Published:** 2023-08-03

**Authors:** Charushila Rukadikar, Chinmay J Shah, Aruna Raju, Sarthak Popat, Rocelyn Josekutty

**Affiliations:** 1 Physiology, All India Institute of Medical Sciences, Gorakhpur, Gorakhpur, IND; 2 Physiology, Government Medical College, Bhavnagar, IND; 3 Physiology, All India Institute of Medical Sciences, Kalyani, Kalyani, IND; 4 Medicine and Surgery, Zydus Medical College & Hospital, Dahod, IND; 5 Medicine and Surgery, Zydus Medical College and Hospital, Dahod, IND

**Keywords:** memory functioning, obesity, cognitive impairments, % body fat, body mass index

## Abstract

Background

Excessive body fat, or obesity, is a worldwide epidemic and a major contributor to the development of dementia.

Aim

The research aimed to determine how obesity affected healthcare professionals' memory performance.

Materials and Method

A total of 474 participants (both male and female) were recruited in this study by random sampling method from three different health institutions. Participants were categorized into overweight, normal weight, and obese groups based on their body mass index (BMI) as per the WHO guidelines and for body fat participants. The memory function test was done using the Gilewski MJ scale. General frequency of forgetting, mnemonic usage, retrospective functioning, and seriousness of forgetting were measured and compared across the BMI and %body fat groups.

Results

The percentage of body fat of males and females was 38.19% and 42.26%. Statistically, a significant difference (p<0.05) was observed among the male and female BMI and percentage of body fat. The results showed that there was a significant difference between memory scale parameters and percentage BMI. Statistically, a significant difference was observed in the level of general frequency of forgetting among participants with different percentages of BMI (p<0.05). Similar, results were also observed in the level of seriousness of forgetting, retrospective functioning, and mnemonics usage with different % BMI (p<0.05). The findings showed a positive correlation between BMI and %body fat on the scale of general frequency of forgetting and seriousness of forgetting whereas, a negative correlation was observed on the scale of retrospective functioning and mnemonics usage.

Conclusion

Memory loss is one of the disorders that obesity is linked to more frequently. A focus on keeping a healthy weight may help prevent the development of future diseases.

## Introduction

The WHO defines obesity and overweight as "excess or abnormal accumulation of fat," both of which may have negative health consequences. A body mass index (BMI) of 25 or above indicates overweight status, whereas a body mass index of 30 or more indicates obesity [1,2]. Overweight and obesity have become a global health crisis due to a doubling in incidence over the last three decades. Overweight prevalence in 2016 was over 1.90 billion, with obesity prevalence at over 650 million. According to the European Health Interview Survey, the rate at which people in Europe are overweight or obese has risen considerably over the last several decades [3]. According to Indian research, the rate of overweight or obese women increased from 20.6 percent in 2016 to 24 percent in 2021. But in 2021, the incidence rate for men increased to around 22.9% [4]. Epidemiological studies have shown a connection between obesity and serious health problems such as diabetes mellitus, cardiovascular disease, musculoskeletal disorders, hypertension, and cancer. The sex of a person influences how their body fat is distributed. Carrying extra weight around the middle is associated with a higher risk of cardiovascular disease, brain atrophy, and cognitive decline in men than in women. Consuming fat and refined meals have been linked to accelerated memory loss and the emergence of neurodegenerative illnesses including dementia and cognitive impairment, in addition to having negative effects on one's physical health [5]. Obesity is linked to cognitive impairments, which are defined as lower-than-normal levels of mental and/or intellectual functioning, according to previous reports [6]. Neuroimaging studies have shown a connection between obesity and both structural and functional changes in the brain, as well as an increased chance of developing Alzheimer's disease (AD). Brain volume, the ability to engage in executive functions, the rate of cognitive growth, and the prevalence of diseases like Alzheimer's have all been shown to be negatively affected by obesity [7,8]. In an 18-year longitudinal study with 1423 participants (61% women), who were in excellent health at the time of cognitive testing, Elias et al. (2015) examined the effects of obesity unrelated to diabetes mellitus (non-insulin-dependent) on cognitive function [9]. According to this study, male obesity had a deleterious impact on cognitive function but not female obesity. Although obese teenagers did not perform worse on cognitive tests than their leaner peers, Yau et al. (2014) discovered evidence of cortical thinning in particular brain regions and decreased microstructural integrity in white matter tracts in these teenagers and linked overall lower academic performance to structural impairments [10]. Prickett et al. (2018) discovered that being overweight predicted worse scores on tests of “verbal memory and working memory”. Results from other neuropsychological examinations have shown similarly poor visual, prospective, and verbal memory abilities [11].

The current study aimed to compare memory performance in people who were either overweight, obese, or of normal weight to better understand the role that body fat, BMI, and different types of memory “short-term memory, non-verbal memory, short-term visual memory, contextual memory, and short-term phonological memory” play in overall memory performance. In this research, we will study the correlation between body mass index, body fat, and memory functioning. 

## Materials and methods

Study design

This study was a cross-sectional multicentric study. After receiving ethical approval from the three institutions i.e., Zydus Medical College & Hospital Dahod, Gujarat, India, India Mahatma Gandhi Medical College and Research Institute, Puducherry, India, Government Medical College, Bhavnagar, Bhavnagar. Gujarat, India, a total of 474 participants from 281 female and 194 male health professionals were recruited. We included participants with informed consent between 18-21 years of age and excluded those who were not interested to participate and who were suffering from diabetes or any other neurological disorders. The sample size is calculated from, the minimum sample size required for accuracy in estimating proportions can be calculated by considering the standard normal deviation set at 95% confidence level (1.96), percentage picking a choice or response (50% = 0.5), and the confidence interval (0.05 = ±5). n=Z_α_^2^pq/e^2, ^z = standard normal deviation set at 95% confidence level, p = percentage picking a choice or response, e is the desired level of precision (i.e. the margin of error).

Participants were asked to fulfill a Gilewski MJ memory function testing questionnaire to measure memory functioning. This is already a standardized and validated questionnaire [12]. The Memory Function Questionnaire (MFQ) was used to examine how patients' self-reported memory functioning compared to their actual performance. According to a four-point Likert scale, respondents are asked to rate how well their memories function on the Memory Functioning Questionnaire (MFQ), with higher scores indicating fewer problems. The following four grading scales are derived from the scores on these items: MFQl = general frequency of forgetting; MFQ2 = seriousness of forgetting; MFQ3 = retrospective functioning, MFQ3 = mnemonics usage. Cronbach's alphas for these scales range from 0.82 to 0.93, which indicates strong internal consistency, as reported by Gilewski et al. (1983) [12]. 

Body Mass Index (BMI) was calculated from the formula “BMI = weight (kg)/ height (m^2^)”. Participants were categorized into overweight, normal weight, and obese groups based on their BMI (BMI <18.5 - underweight, 18.5-<25 - healthy weight, 25-<30 - overweight, >30 - obese).

The amount of body fat was measured by measuring the thickness of skin folds at four different sites viz bicep, triceps, supra-iliac, and subscapular. A skin fold caliper was used to measure skin fold thickness. The caliper was calibrated before use. The participant was instructed to extend the arm for measurement of bicep skin fold thickness. the measurement taken on the skin midway between the shoulder and elbow on the front of the arm with the caliper held perpendicularly to the skin [13]. The participant was instructed to stand straight with their arm hanging by the side for measurement of tricep skin fold thickness. Measurement was taken on the back of the arm, midway between the shoulder and elbow. The participants were instructed to stand up straight with their feet shoulder-width apart for measurement of supra iliac skin fold thickness. The skin fold caliper was kept at the point of the hip bone on the side of the body, approximately two centimeters above the iliac crest. The caliper was placed at a 45-degree angle to the skin fold, and the measurement was taken at the mid-point of the fold. The participant was instructed to stand up straight with their arms relaxed at their sides for measurement of subscapular skin fold thickness. The skin fold thickness was measured at a diagonal line, one centimeter below the lower angle of the scapula. The caliper is placed perpendicular to the skin fold, and the measurement should be taken at the mid-point of the fold [14]. Body density values were calculated using the specific equation of “Durnin and Womersley” Men: D (g cm3) =1:1765 − 0:0744 log10ð Þ Σ4SF & Women: D (g cm3) =1:1339 − 0:0645 log10ð Þ. The body density equations were converted into fat percentages, by using the “Siri equation”: %BF = ((4.95/D) - 4.50) × 100 [14] based on body fat %, participants were categorized into athletes: 14-20% (women), 6-13% (men), fitness: 21-24% (women), 14-17% (men), average: 25-31% (women), 18-24% (men), and obese: 32%+ (women), 25%+ (men).

Statical analysis

The SPSS (IBM Corp. Released 2017. IBM SPSS Statistics for Windows, Version 25.0. Armonk, NY: IBM Corp) software was used to perform the statistical tests. The mean and standard deviation were used to describe all continuous values, whereas percentages and numbers were used to represent categorical data. Descriptive statistics were used to determine the frequencies and proportions. Analysis of variance formula (ANOVA) was used to determine the significant difference whereas regression was used to measure the correlation. 

## Results

Table 1 showed among the 474 participants, 194 were male, and 281 were female. The BMI of males and females was observed at 27.15 and 35.41 respectively. The % of body fat of males and females was 38.19 and 42.26. Statistically, a significant difference (p<0.05) was observed among the male and female BMI and % body fat.

**Table 1:** Descriptive statistics of male and female BMI and % body fat BMI - Body mass Index, * = Statastically significant

Variables	Male (n=194)		Female (n=281)		P value
	Mean±SD	Range	Mean±SD	Range	
BMI (kg/m^2^)	27.15±3.8	16.94- 28.26	35.41±4.82	28.14-36.19	0.0001*
% Body Fat	38.19±5.19	26.33-42.19	42.26±3.44	34.85-56.72	0.0001*

The distribution of the participants' memory functioning questionnaire scores based on their BMI were represented in Table 2. The majority of the participants were of normal BMI (BMI= 18.5-<25), followed by underweight (BMI <18.5) then overweight (BMI= 25-<30), and 58 of them were considered obese (BMI>30). A higher rate of general frequency of forgetting, the seriousness of forgetting, retrospective functioning, and mnemonics use was observed in obese participants. 

**Table 2:** Statistics of memory functioning questionnaire on the function of BMI * = Statastically significant (p value), Degrees of freedom (df), sum-of-squares (SS), Mean squares (ms), F ratio (F), Coeff. = Coefficient

General Frequency of Forgetting					Seriousness of forgetting				Retrospective functioning				Mnemonics Usage			
	df	SS	MS	F	df	SS	MS	F	df	SS	MS	F	df	SS	MS	F
Regression	1	869.18	869.18	26.80	1	311.96	311.96	9.28	1	845.93	845.93	26.04	1	569.8	569.8	17.23
Residual	472	15306.1	32.42		472	15863.39	33.60		472	15329.42	32.47		472	15605.5	33.06	
Total	473	16175.3			473	16175.35			473	16175.35			473	16175.3		
	Coeff.	Standard Error	t Stat	P-value	Coeff.	Standard Error	t Stat	P-value	Coeff.	Standard Error	t Stat	P-value	Coeff.	Standard Error	t Stat	P-value
Intercept	29.39	1.2	23.1		25.8	0.97	26.44		27.64	0.954	28.95		27.07638	1.02	26.38	
X Variable 1	-0.04	0.007	-5.1	0.004*	-0.03	0.01	-3.046	0.002*	-0.2	0.04	-5.10	0.001*	-0.135	0.03	-4.15	0.003*

Table 3 represents the ANOVA statistics of memory functioning of % BMI. The results showed that there was a significant difference between memory scale parameters and % BMI. Statistically, a significant difference was observed in the level of general frequency of forgetting among participants with different % BMI (p<0.05). Similar, results were also observed in the level of seriousness of forgetting, retrospective functioning, and mnemonics usage with different % BMI (p<0.05).

**Table 3:** Statistics for memory functioning questionnaire scores as function of % body fat

Group	Body fat % For male	Body fat % for female		General Frequency of Forgetting	Seriousness of forgetting	Retrospective functioning	Mnemonics Usage
			n	M±SD	M±SD	M±SD	M±SD
Athletes	6-13%	14-20%	15.00	158.40±34.37	80.46±29.19	26.13±5.40	31.53±8.21
Fitness	14-17%	21-24%	48.00	164.08± 38.00	82.83±26.38	16.52±0.81	30.91±8.81
Acceptable	18-25%	25-31%	274.00	162.12± 31.62	86.01±24.22	23.48±6.71	31.37±8.77
Obese	>25%	>32%	137.00	140.97± 28.44	73.82±16.59	20.54±5.04	28.01±5.65

Table 4 showed statistics for memory functioning questionnaire score as a function of body fat %. The obese group had more values of mean and standard deviation (SD) in the general frequency of forgetting, the seriousness of forgetting, retrospective functioning, and mnemonics usage.

**Table 4:** T-test and ANOVA statistics of memory functioning questionnaire on function of BMI

General Frequency of Forgetting					Seriousness of forgetting				Retrospective functioning				Mnemonics Usage			
	df	SS	MS	F	df	SS	MS	F	df	SS	MS	F	df	SS	MS	F
Regression	1	869.18	869.18	26.80	1	311.96	311.96	9.2822	1	845.9314	845.9314	26.04662	1	569.8149	569.8149	17.23444
Residual	472	15306.16	32.42		472	15863.39	33.60		472	15329.42	32.47759		472	15605.54	33.06258	
Total	473	16175.35			473	16175.35			473	16175.35			473	16175.35		
	Coefficients	Standard Error	t Stat	P-value	Coefficients	Standard Error	t Stat	P-value	Coefficients	Standard Error	t Stat	P-value	Coefficients	Standard Error	t Stat	P-value
Intercept	29.39218	1.2695	23.150	8.68E-80	25.822	0.9764	26.44	3.28E-95	27.64	0.954	28.95893	9.7E-107	27.07638	1.026068	26.38848	6.02E-95
X Variable 1	-0.0412	0.0079	-5.177	3.34E-07	-0.034	0.0114	-3.046	0.0024	-0.205	0.040	-5.103	4.84E-07	-0.135	0.032659	-4.151	3.92E-05

Table 5 represents the statistics of memory functioning of % body fat. The results showed that there was a significant difference between memory scale parameters and % body fat. Statistically, a significant difference was observed in the level of general frequency of forgetting among participants with different % body fat (p<0.05). Similar, results were also observed in the level of seriousness of forgetting, retrospective functioning, and mnemonics usage with different % body fat (p<0.05).

**Table 5:** Statistics of memory functioning of % body fat * = statistically significant, Degrees of freedom (df), sum-of-squares (SS), Mean squares (ms), F ratio (F), Coeff. - Coefficient

General Frequency of Forgetting					Seriousness of forgetting				Retrospective functioning				Mnemonics Usage			
	df	SS	MS	F	df	SS	MS	F	df	SS	MS	F	df	SS	MS	F
Regression	1	2239.7	2239.7	84.58	1	761.5	761.5	25.71	1	1228.6	1228.6	42.9	1	611.3	611.3	20.4
Residual	472	12498.4	26.4		472	13976.7	29.61		472	13509.6	28.6		472	14126.9	29.9	
Total	473	14738.2			473	14738.2			473	14738.2			473	14738.2		
	Coeff.	Standard Error	t Stat	P-value	Coeff.	Standard Error	t Stat	P-value	Coeff.	Standard Error	t Stat	P-value	Coeff.	Standard Error	t Stat	P-value
Intercept	33.7	1.1	29.41		27.89	0.91	30.4		29	0.89	32.4		27.68347	0.97	28.35697	
X Variable 1	-0.06	0.007	-9.1	0.003*	-0.05	0.01	-5.07	0.004*	-0.2	0.03	-6.5	0.005*	-0.14043	0.03	-4.51939	0.005*

Table 6 represents the correlation of memory functioning with BMI and body fat with regression factor. The findings showed a positive correlation between BMI and %body fat on the scale of general frequency of forgetting and seriousness of forgetting whereas, a negative correlation was observed on the scale of retrospective functioning and mnemonics usage.

**Table 6:** Regression statistics of memory functioning questionnaire

Regression Factors	General Frequency of Forgetting	Seriousness of forgetting	Retrospective functioning	Mnemonics Usage
General Frequency of Forgetting	0.84	0.64	0.59	0.38
Seriousness of forgetting	0.62	0.71	0.48	-0.67
Retrospective functioning	0.63	-0.52	-0.26	-0.55
Mnemonics Usage	0.30	0.39	-0.76	0.50

## Discussion

The occurrence of obesity is increasing worldwide, and this condition is linked to several diseases. Obesity-related behaviors including eating habits and lack of exercise have also been linked to an increased risk of developing diseases like Alzheimer's disease. Obesity may have a negative impact on memory function, which involves recalling and reliving moments or events in time and space [15]. Our findings imply that worse memory function in obese people was associated with a higher BMI. Additionally, people with higher BMI were shown to have a worse working memory, which is defined as the capacity to manage and temporarily retain data to carry out complicated cognitive activities. The findings of our study are consistent with other research suggesting that a higher BMI commonly has an impact on working memory. Working memory has been characterized as the capacity to retain knowledge in the mind. The findings support the hypothesis that obesity and overweight contribute to the cognitive decline of working memory. Our findings emphasize the need of examining the diverse roles of body fat and BMI in the cognitive function of people with overweight and obesity since the accumulation of fat in different parts of the body might indicate different profiles of cognitive impairment. Since it has been shown that BMI has a significant effect on memory, it is crucial to investigate how this has an impact on certain memory domains. We found a stronger correlation between BMI and % of body fat on the memory functioning scale. In previous research by Nyberg et al. (2020), it was discovered that having a greater body fat percentage was associated with smaller subcortical and hippocampus volumes and worse memory. They also demonstrated that memory functions have a significant impact on the regulation of appetite and the storage of fat. Interfering with memory processes may result in overeating and obesity because the remembered experience is more strongly related to subsequent choices than the actual occurrence [15-16]. Participants with normal and excessive weight exhibited identical findings for memory tasks, except for “short-term phonological memory and working memory”, where those with obesity performed worse. According to research by Gunstad et al. (2006), obese persons had inferior memory test results when compared to adults of all ages (adults aged 21 to 82), as well as when just looked at younger and middle-aged adults (adults aged 21 to 50). Regression studies revealed no indication of an age-BMI interaction on any memory test, proving that the link between BMI and memory is age-independent. These data imply that these effects are not only restricted to older persons and further confirm the independent link between obesity and worse memory function. To pinpoint the etiological elements, further investigation is required [17]. The attention domain includes four tests that Gunstad et al. (2007) found no significant differences between normal weight and overweight/obese people on switching of attention, digit span forward, choice response time, and span of visual memory [18]. According to studies by Medawar et al., 2022, people who have a higher BMI and more visceral fat also tend to have more subtle grey matter atrophy and white matter lesions, a different hypothalamic microstructure, and decreased thickness, memory, and connectivity in reward and default mode network regions. Analysis of mediation showed that localized anomalies linked to obesity result in cognitive deficits and that increasing visceral fat adversely affects brain tissue via systemic low-grade inflammation [19]. Similarly, memory and neuroanatomical assessments were shown to be generally declining in our study. According to Bilbo and Tsang's study, young adult female and male rats that were born to dams who were given a diet rich in saturated or trans fats outperformed control rats by a large margin in the Morris water maze challenge. Offspring of obese peromyscus mice at three months of age, and piglets at 11-15 weeks of age who were fed high-fat diets prenatally or both prenatally and postnatally all showed enhanced spatial memory ability [20]. Similar conflicting results have been reported by Graf et al., 2016 when employing the new object identification test. In one experiment, young adult male-but not female-offspring of fat dams showed lower exploration of the novel item [21]. Shen et al., 2019 found that obese obstructive sleep apnea (OSA) patients showed slower response times in the psychomotor vigilance test but not in the Flanker or Stroop cognition tasks as compared to non-obese OSA patients. Furthermore, obese OSA patients exhibited worse working memory ability than non-obese OSA patients [22]. Verstraeten et al. found that OSA patients had decreased visual alertness, which was characterized by attention lapses and state instability. However, the therapy benefit of continuous positive airway pressure in OSA patients has received little attention [23]. According to Nyberg et al., 2020, greater body fat has a detrimental influence on memory deterioration in cognitively healthy individuals, with the effect increasing with age for the neuroanatomical volumes. Similar conflicting results our findings revealed that body fat was adversely associated with memory [16]. Fjell et al. (2015) investigated the link between body fat to learn more about the potential impact of obesity on memory performance at the brain level. Body fat had a negative correlation with volume change that was significantly impacted by age, with body fat having a less negative correlation as one becomes older. No age interaction was seen in the relationship between body fat and memory [24]. Research by Khan et al., 2014 revealed that pre-existing or post-obesity primed memory CD8 T cell responses are unaffected by the obese environment. This finding has surprising but important implications for future research into the role obesity plays in host susceptibility to infections [25]. Berbegal et al., 2022 found that those who were considered obese had worse levels of “short-term phonological memory and working memory” compared to people of normal weight. Memory performance was negatively related to body mass index, waist-hip ratio, total fat, and visceral fat, according to bivariate correlations. On the other hand, the correlation between muscular mass and cognitive performance was weak. Memory impairment was also associated with greater systolic blood pressure. This research demonstrates the significance of body fat on health and cognitive performance [26].There is evidence that biological sex affects memory function in a significant way, with each category of memory being affected differently by sex. Research explore putative mechanisms, such as sex-specific changes in neuroanatomy, neurochemistry, biology, cognition, and affect, that could account for those sex-specific effects [27].

Study Limitations

Self-Reporting Bias

The study's evaluation of obesity and cognitive function may have relied on self-reported data. Self-reported information may be subject to recall bias or social desirability bias, which can result in inaccurate or lacking information.

Confounding Factors

The study does not have taken into account all possible confounding factors that could affect the association between obesity and cognitive performance. The accuracy of the results may be hampered by the exclusion of variables like medication use because these variables may have an impact on both obesity and cognitive function.

Lack of Objective Measurements

Without objective evaluations or standardized tests, the study may have only used self-reported measures of cognitive functioning. The accuracy and robustness of the study's conclusions may be impacted by the absence of objective measurements because they produce more valid and reliable data.

Absence of Longitudinal Follow-up

Longitudinal studies that keep track of participants over time are better suited to investigate the long-term effects of obesity on cognitive functioning. The study has difficulty capturing the dynamic nature of the relationship if it lacked a longitudinal design or a follow-up element.

External Validity

Healthcare professionals have distinctive traits, work-related factors, and lifestyles that are different from those of the general population, which restricts the generalizability of the findings.

## Conclusions

The epidemic of obesity in the modern world is a serious threat to people's health and well-being on a societal and personal level. Particularly relevant are the cognitive effects of being overweight. Our findings imply an association between obesity and poor memory function. Participants' memory capacity was reduced and their response times were slower on the psychomotor vigilance test when they were obese. If the present obesity trend continues, chronic illness will become epidemic in coming generations and lead to global health and economic calamity. Increasing your fat and/or sugar consumption may be detrimental to your memory in a variety of ways. To understand the pathophysiology and etiology of obesity in the future, it will be necessary to isolate the main genes involved. Effective initiatives that effectively encourage greater physical activity and good eating across communities are still needed to avoid obesity and the illnesses it is connected with; this will need the active engagement of both people and their governments. More research is required to determine the most effective therapeutic targets and treatment combinations to guarantee long-term health benefits. 

Recommendations

Healthcare personnel have a critical obligation to lead healthy lifestyles as role models for their patients. By setting an example for others, leaders establish their own credibility and show how important it is to lead by example. Healthcare practitioners can be a source of motivation and inspiration for patients looking to make healthy lifestyle changes by adopting good eating practices, participating in regular physical activity, and managing stress in an efficient manner. Additionally, healthcare providers should stay current on the most recent findings and recommendations regarding obesity and mental health. With this information, they will be better equipped to advise patients on dietary changes and physical activity modifications that can lessen the detrimental consequences of obesity on cognition. Collaboration amongst researchers from diverse fields is necessary to advance our understanding of the relationship between obesity and cognitive function. A comprehensive understanding of the complex issues of obesity-related cognitive impairment can be obtained by working with specialists in the fields of neuroscience, psychology, genetics, nutrition, and public health. Finding fundamental mechanisms and viable treatment approaches can advance with the sharing of data, methodology, and insights. International collaboration can also provide important insights into how various social, environmental, and cultural factors affect the association between obesity and cognition. By combining their resources and knowledge, researchers can carry out extensive studies that result in more solid and broadly applicable conclusions. These joint initiatives will help build a substantial body of research that may guide public health policies and actions designed to counter the obesity pandemic and its effects on cognition.
